# Knowledge about childhood autism among health workers (KCAHW) questionnaire: description, reliability and internal consistency

**DOI:** 10.1186/1745-0179-4-17

**Published:** 2008-06-06

**Authors:** Muideen O Bakare, Peter O Ebigbo, Ahamefule O Agomoh, Nkem C Menkiti

**Affiliations:** 1 Child and Adolescent Unit, Federal Neuro-Psychiatric Hospital, New Haven, Enugu, Enugu State, Nigeria; 2 Department of Psychological Medicine, University of Nigeria Teaching Hospital (UNTH), Enugu, Enugu State, Nigeria; 3 African Network for the Prevention and Protection against Child Abuse and Neglect (ANPPCAN), Nigeria; 4 International Federation for Psychotherapy Centre, Uwani, Enugu, Enugu State, Nigeria

## Abstract

**Background:**

Knowledge and awareness about childhood autism is low among health workers and the general community in Nigeria and other Sub-Saharan African countries. Poor knowledge and awareness about childhood autism, especially among health workers can compromise early recognition and interventions which had been known to improve prognosis in children with autism. In formulating policy and designing interventions for these children, there is need to develop a reliable tool that can be used in assessing baseline knowledge about childhood autism among health workers and the impact that future continued education and awareness campaign may have on such baseline knowledge. Knowledge about childhood autism among health workers (KCAHW) questionnaire was designed for this purpose.

**Methods:**

The KCAHW questionnaire is a nineteen (19) item self-administered questionnaire that is divided into four domains. KCAHW questionnaires were distributed to fifty (50) psychiatric nurses involved in community mental health services in South-Eastern Nigeria to complete. After two weeks period, the KCAHW questionnaires were re-administered to the same fifty (50) psychiatric nurses to assess their knowledge about childhood autism and to assess the test-retest reliability and internal consistency of this questionnaire.

**Results:**

KCAHW questionnaire showed good test-retest reliability when the mean domain and total scores at first and second time administration were compared. The four mean domain scores and the mean total scores at first and second time administration were significantly correlated. The questionnaire also had a good overall internal consistency when the mean scores of the four domains were correlated with mean total scores (Cronbach's alpha = 0.97).

**Conclusion:**

The KCAHW questionnaire is a reliable tool for assessing knowledge of health workers about childhood autism. It would be a useful tool in improving early recognition of features of autism among affected children in Sub-Saharan African and other developing countries of the world where knowledge and awareness about childhood autism is low.

## Background

Since Leo Kanner's paper titled, "Autistic disturbances of affective contact" about seven decades ago [1], awareness and further research about childhood autism have continued to grow globally. There had been observation that the prevalence of childhood autism and Autistic Spectrum Disorder (ASD) were on the increase worldwide [2-8]. This prevalence increase had also been thought to be attributable to increased knowledge and awareness among health workers and possible adoption of broader criteria in making the diagnosis [9]. While knowledge and research on childhood autism and ASD are on the increase in parts of the world, knowledge and research about this condition are limited in Nigeria and other Sub-Saharan African countries [10]. Possibly because of the limited research and knowledge about childhood autism in Sub-Saharan Africa, the occurrence of this condition among Africans and universality of childhood autism had been queried [11]. However, there is no doubt that childhood autism occurs among Africans [12-15]. The recognition among health workers in this region may just be low because of low level of awareness and knowledge. Also, childhood autism and other related syndromes associated with mental retardation rarely comes to the attention of clinician in Nigeria possibly because of discrimination and stigmatization associated with the etiological explanation of these syndromes which in turn influence the help seeking behavior[16]. The evidence of low level of awareness and rare presentation to clinicians in this part of the world can be seen in the reported cases from Nigeria where the patients were coming in contact with orthodox care for the first time as adolescents [14,15].

### Knowledge about childhood autism in health care settings

Earlier survey on knowledge about childhood autism among health care workers had been by Stone [17]. The study found that the specialists' views on childhood autism were consistent with the prevalent views in research literature then, but it was also found that individual disciplines studied displayed variation and misconceptions regarding social, emotional and cognitive aspects of autism. Diagnostic criteria were found to also differ among the groups of health care workers studied. Heidgerken et al [18] also did a relatively recent survey of knowledge about autism in health care settings, among professionals from the Center for Autism Related Disabilities (CARD), specialists like psychiatrists, speech and language pathologists, clinical psychologists, and primary health care providers like family physicians, pediatricians, and neurologists. They used a measure that assessed knowledge of diagnostic criteria, course, treatment and prognosis of autism. Their findings indicated that all three groups of CARD professionals, specialists and primary health care providers reflected accurate endorsement of the DSM-IV diagnostic criteria for autism. Primary health care providers and specialists were found to differentially endorse a variety of statements about prognosis, course and treatment when compared with CARD group. The study concluded that primary health care providers showed greatest number of differences among the three groups. In a more recent survey of knowledge about autism among speech-language pathologists, Schwartz and Drager [19] noted that most participants in their study had accurate knowledge about the characteristics of children with autism. However, it was noted that these participants had mixed perceptions of diagnostic criteria for autism and reported that they could have benefited from additional training in the area of autism.

Rhoades et al [20] explored the importance of physicians' knowledge of autism spectrum disorders (ASD) and found that physician's knowledge or lack of knowledge about autism greatly influenced the average age of diagnosis of ASD, which is important to ultimate prognosis and also whether the physician provides further information necessary to care-givers about autism or not.

The above surveys coming from the United States of America (USA), an environment where knowledge and research about childhood autism are more widely spread than what obtains in sub-Saharan Africa point to the need to assess knowledge and awareness in this environment and put in place programs to increase such knowledge and awareness with the ultimate aim of helping children with ASD.

It had been presumed that in view of the rarity of presentation of childhood autism cases in pediatric health facilities in developing countries especially Sub-Saharan Africa, health care workers especially the nurses who are often in charge of various Primary Health Care (PHC) facilities in rural communities in Africa might not be well versed with the condition. Subsequently, early identification at the community level and intervention which have been shown to improve the prognosis of these cases [8,21,22] may be compromised.

This presumption was put into test in May 2007, when African Network for the Prevention and Protection Against Child Abuse and Neglect (ANPPCAN), Nigeria Chapter in collaboration with the World Bank carried out a survey to ascertain the level of knowledge and awareness of health care workers and the general public in Enugu State, South Eastern Nigeria on autism and implemented a project aimed at increasing the level of awareness on autism.

The results of the survey showed that there is a very low level of awareness on autism among the general public and a low to average level of awareness among various categories of health care workers, with highest level of awareness noted among those health workers working in psychiatric facilities. Attempt was made then, to increase the level of awareness on autism through a workshop organized for the stakeholders and a radio phone – in program for the general public. However, one of the problems encountered during the survey was the unavailability of easily understandable and reliable questionnaire for measurement of the health care workers baseline knowledge of autism, aimed at early identification of cases in the community [23]. The KCAHW questionnaire is a modified version of an earlier questionnaire used among health care workers in the described survey which had been revised with removal and addition of some items based on the problems encountered during the earlier survey carried out by ANPPCAN, Nigeria Chapter in collaboration with the World Bank [23].

It had therefore been established that knowledge and awareness on autism and ASD is low in south-eastern Nigeria and there had been recent outcry in Nigeria to promote knowledge and awareness about childhood autism among the health care workers and the general community [24].

In doing this, there is need for baseline assessment of present knowledge, facilities in place for intervention among other things. This would ensure proper planning. Promoting awareness at the community level should begin with various health workers who are involved in community health services. They can be trained to become trainers of other category of people in the community towards early recognition of children with childhood autism.

For proper design of community campaign and continued education needed for these health workers, the need arises to design a reliable tool that can be used in measuring their baseline knowledge about childhood autism and the possible impact that future continued education and campaign may have on the knowledge of these health workers about childhood autism.

Knowledge and awareness about childhood autism may vary from one geographical region to another. The same tool can be used to assess variation in knowledge of the disease condition over different geographical regions, given an index of geographical locations where immediate attention need to be focused as regards health workers and community education.

The above reasons inform the design of *"Knowledge about Childhood Autism among Health Workers (KCAHW) Questionnaire" *for use in various communities of developing countries where knowledge about childhood autism is limited among the health workers working at the community level.

This article is aimed at describing the content of KCAHW questionnaire and reporting the test-retest reliability and internal consistency of the questionnaire.

## Methods

Location of the study was at Federal Neuro-Psychiatric Hospital, New Haven, Enugu (FNHE), Nigeria. FNHE is a specialist Psychiatric Hospital located in the South-Eastern region of Nigeria. The hospital is involved in community mental health service collaboration with various PHC facilities located in the rural communities of South-Eastern Nigeria. This community mental health care service in various PHC facilities in this region of the country is overseeing by the psychiatric nurses employed by the hospital.

Ethical approval for this study was obtained from the ethical committee of FNHE, Nigeria.

A total of fifty (50) psychiatric nurses employed by FNHE, Nigeria and who are involved in the community mental health services of the hospital participated in the study. There were 12 males and 38 females. The fifty nurses included in the study had had a minimum of five years of practice in general psychiatry nursing and possessed diploma qualification in general nursing and psychiatry nursing. It should be noted that only psychiatric nurses were used as the sample for this study because in the context of our environment in Nigeria and other sub-Saharan African countries, they are supposed to be the most likely health care workers who attend to cases such as autism and ASD and still supervise health care services at primary care level.

In addition to the socio-demographic questionnaire, KCAHW questionnaire was distributed to consented fifty (50) psychiatric nurses to complete. After a period of two (2) weeks, the KCAHW questionnaire was re-distributed to the same fifty (50) psychiatric nurses to complete. The questionnaires were completed by the respondents and collected from them there and then at each point of administration to prevent them from consulting any study material or discussing with their colleagues before filling in their responses.

### KCAHW Questionnaire (Appendices 1 and 2)

This is a self administered questionnaire that contained a total of nineteen (19) – item questions. Each of the item questions has three (3) options to choose from with only one of these three options being correct. The correct option on each item question attracts a score of one (1), while the other two options that are incorrect attract a score of zero (0) each.

The KCAHW questionnaire is further divided into four (4) domains:

#### Domain 1

Contained eight (8) item questions that addressed the impairments in social interaction usually found in children with childhood autism. A maximum and minimum score of 8 and 0 respectively are possible in this domain.

#### Domain 2

Contained only one (1) item question that addressed impairment in area of communication and language development, which is part of symptom presentation in children with childhood autism. A maximum and minimum score of 1 and 0 respectively are possible in this domain.

#### Domain 3

Contained four (4) item questions that addressed area of obsession and compulsive pattern of behavior found in children with childhood autism, a pattern of behavior which had been described as restricted, repetitive and stereotyped. A maximum and minimum score of four (4) and zero (0) respectively are possible in this domain.

#### Domain 4

Contained six (6) item questions that addressed information on what type of disorder is childhood autism, possible co-morbid conditions and onset of childhood autism in affected children. A maximum and minimum score of six (6) and zero (0) respectively are possible in this domain.

Therefore, a maximum and minimum total score of nineteen (19) and zero (0) respectively are possible when the four domain scores are added together.

The content of the questionnaire and the scoring system are shown in Appendices 1 and 2.

The mean total score on KCAHW questionnaire among a particular sample population or community is a measure of level of knowledge about childhood autism among that particular sample population or community.

### Data Analysis

The mean scores in each domain and the mean total scores generated when KCAHW questionnaires were administered the first and second time were compared with paired t-test statistical analysis to assess the test-retest reliability of the KCAHW questionnaire.

Then, the mean inter-domain scores during the first and second time administration of the questionnaire were correlated with the mean total scores and cronbach's alpha [25] was calculated for the correlations to assess the internal consistency of KCAHW questionnaire.

## Results

A total of fifty (50) psychiatric nurses employed by FNHE, Nigeria and who are involved in the community mental health services of the hospital participated in the study. There were 12 males and 38 females with mean age of 32.6 years. The mean scores in the four domains of KCAHW questionnaire and mean total scores of 9.6 and 9.8 on first and second time administration of KCAHW questionnaire respectively which gave indices of level of knowledge on childhood autism in that particular sample population are shown in Table 1.

**Table 1:** Mean scores in the four domains and mean total scores on KCAHW questionnaire at first and second time of KCAHW questionnaire administration

Domains	(A&B)	Mean	Standard Deviation	Standard Error
Domain 1	Domain 1A Vs Domain 1B	4.60 4.60	2.52 2.52	0.36 0.36
Domain 2	Domain 2A Vs Domain 2B	0.60 0.80	0.50 0.40	0.07 0.06
Domain 3	Domain 3A Vs Domain 3B	2.00 1.80	1.43 1.34	0.20 0.19
Domain 4	Domain 4A Vs Domain 4B	2.40 2.60	1.37 0.81	0.19 0.11
Total	Total A	9.60	5.25	0.74
	Total B	9.80	4.67	0.66

A – First time administration of KCAHW questionnaire

B – Second time administration of KCAHW questionnaire

The paired t-test analysis comparing these mean scores and the 95% Confidence Interval (95% C.I) for the differences in the mean scores are shown in Table 2.

**Table 2:** Paired t-test table comparing the mean scores in the four domains and mean total scores on KCAHW questionnaire at first and second time of KCAHW questionnaire administration

Domains	A&B	Paired Mean Difference	Stand. Dev.	Stand. Error Mean	95% C.I Lower	95% C.I Upper	t	df	Sig (2tailed)
Domain 1	A & B	0.00	0.64	0.09	- 0.18	0.18	0.00	49	1.00
Domain 2	A & B	-0.20	0.40	0.06	-0.32	-0.09	-3.50	49	0.00
Domain 3	A & B	0.20	0.40	0.06	0.09	0.32	3.50	49	0.00
Domain 4	A & B	-0.20	0.76	0.11	-0.42	0.02	-1.87	49	0.07
Total	A & B	-0.20	0.76	0.11	-0.42	0.02	-1.87	49	0.07

A – First time administration of KCAHW questionnaire

B – Second time administration of KCAHW questionnaire

The correlation co-efficient for the four mean domain scores and mean total scores at first and second time administration of KCAHW questionnaire are shown in Table 3. The values showed significant correlations both in the mean domain scores and mean total scores.

**Table 3:** Paired t-test showing mean domain scores and mean total scores correlation co-efficient

Domains	(A & B)	Correlation Co-efficient	Sig.
Domain 1	A & B	0.97	0.000
Domain 2	A & B	0.61	0.000
Domain 3	A & B	0.96	0.000
Domain 4	A & B	0.89	0.000
Total	A & B	0.99	0.000

A – First time administration of KCAHW questionnaire

B – Second time administration of KCAHW questionnaire

The internal consistency of KCAHW questionnaire was assessed by correlating different mean domain scores with the mean total scores at first and second time administration of the questionnaire and calculating the cronbach's alpha. The cronbach's alpha value of 0.97 obtained following the correlation is acceptable based on Nunnaly's recommendation [26].

The ten by ten correlation matrix table of the four mean domain scores and mean total scores at first and second time administration of the KCAHW questionnaire is shown in Table 4.

**Table 4:** Correlation matrix table showing the internal consistency of KCAHW questionnaire

Correlation Matrix										
	**D 1A**	**D 1B**	**D 2A**	**D 2B**	**D 3A**	**D 3B**	**D 4A**	**D 4B**	**T A**	**T B**

D 1A	1.0000									
D 1B	0.9679	1.0000								
D 2A	0.3595	0.5230	1.0000							
D 2B	0.9207	0.9207	0.6124	1.0000						
D 3A	0.7926	0.7360	0.0000	0.7071	1.0000					
D 3B	0.7604	0.6397	-0.1231	0.6784	0.9594	1.0000				
D 4A	0.9916	0.9326	0.2408	0.8847	0.8341	0.8224	1.0000			
D 4B	0.9207	0.9207	0.6124	1.0000	0.7071	0.6784	0.8847	1.0000		
T A	0.9885	0.9577	0.3297	0.9231	0.8703	0.8292	0.9867	0.9231	1.0000	
T B	0.9805	0.9632	0.4063	0.9520	0.8568	0.8090	0.9698	0.9520	0.9953	1.0000

Number of Cases = 50.0

Reliability Coefficients – 10 items

Alpha = 0.92 Standardized item alpha = 0.97

D – Domains, T – Total, A – First time administration of KCAHW questionnaire

B – Second time administration of KCAHW questionnaire

## Discussion

The content of KCAHW questionnaire had been described. The test-retest reliability of the questionnaire is good based on the significant correlations in the mean domain and mean total scores at first and second time of administration. The KCAHW questionnaire also showed a good internal consistency as revealed in the cronbach's alpha value obtained for mean domain scores correlation with the mean total scores both at first and second time of administration.

The imperative need to increase the level of knowledge and awareness among health workers and of the general community in Sub-Saharan African and other developing countries of the world about childhood autism necessitated the design of a reliable measuring tool that can be used to assess baseline knowledge of health workers on childhood autism and also subsequently the impact the future campaign and continuous education may have on the knowledge of these health workers about childhood autism, especially those group of health workers at primary health care facilities who are closer to the communities.

KCAHW questionnaire could also be used to compare knowledge about childhood autism among different categories of health workers, if there is an indication for such. The variation in knowledge about childhood autism among health workers that could exist in various geographical regions of Sub-Saharan African and other developing countries where awareness about autism and ASD is low could also be assessed with KCAHW questionnaire.

## Limitations

The limitation of administering the KCAHW questionnaire is that it could only be completed and collected immediately from the respondents upon administration. This is to prevent consultation of study materials or discussion with colleagues which can influence the respondents' response to the questions contained in the questionnaire. The KCAHW questionnaire would therefore serve the purpose only of a point time assessment of knowledge.

## Conclusion

KCAHW questionnaire is a reliable tool for assessing knowledge of health workers about childhood autism, aimed at early recognition of the condition in the primary care settings. It would be a valuable tool in assessing baseline knowledge of health workers about childhood autism in Sub-Saharan African communities and other developing countries where public awareness about childhood autism is low and policy and interventions are being planned for children affected by childhood autism. The level of knowledge of health workers about childhood autism, especially those working in the rural communities in this environment and other developing countries is an essential ingredient for early recognition and intervention which had been noted to improve prognosis in the affected children [8,17,18]. KCAHW questionnaire would be a useful tool in Sub-Saharan African and other developing countries where knowledge and awareness about childhood autism is low.

## Competing interests

POE is affiliated with African Network for the Prevention and Protection against Child Abuse and Neglect (ANPPCAN), Nigeria Chapter and ANPPCAN, Nigeria Chapter received sponsorship from the World Bank in May 2007 for a survey on level of awareness of autism and implementation of program to raise awareness on autism in Enugu State, South Eastern Nigeria.

## Authors' contributions

All authors contributed to the conception of the study. MOB, POE and AOA are involved in writing and revision of the manuscript. All authors approved the final draft of the manuscript.

## Appendix 1

### Knowledge about Childhood Autism among Health Workers (KCAHW) Questionnaire

Please do not consult formal text books to answer these questions.

Thank you for your time.

The following behaviors best describe a child with Childhood Autism:

#### Domain 1

i. Marked impairment in use of multiple non-verbal behaviors such as eye to eye contact, facial expression, body postures and gestures during social interaction?

(A) Don't Know, (B) Yes, (C) No

ii. Failure to develop peer relationship appropriate for developmental age?

(A) Don't Know, (B) Yes, (C) No

iii. Lack of spontaneous will to share enjoyment, interest or activities with other people? (A) Don't Know, (B) Yes, (C) No

iv. Lack of social or emotional reciprocity? (A) Don't Know, (B) Yes, (C) No

v. Staring into open space and not focusing on any thing specific?

(A) Don't Know, (B) Yes, (C) No

vi. The child can appear as if deaf or dumb? (A) Don't Know, (B) Yes, (C) No

vii. Loss of interest in the environment and surroundings?

(A) Don't Know, (B) Yes, (C) No

viii. Social smile is usually absent in a child with Autism?

(A) Don't Know, (B) Yes (C) No

#### Domain 2

i. Delay or total lack of development of spoken language?

(A) Don't Know (B) Yes (C) No

#### Domain 3

i. Stereotyped and repetitive movement (e.g. Hand or finger flapping or twisting)?

(A) Don't Know (B) Yes, (C) No

ii. May be associated with abnormal eating habit?

(A) Don't Know, (B) Yes, (C) No

iii. Persistent preoccupation with parts of objects?

(A) Don't Know, (B) Yes, (C) No

iv. Love for regimented routine activities? (A) Don't Know, (B) Yes, (C) No

#### Domain 4

i. Autism is Childhood Schizophrenia? (A) Don't Know, (B) Yes, (C) No

ii. Autism is an auto-immune condition? (A) Don't Know, (B) Yes, (C) No

iii. Autism is a neuro-developmental disorder? (A) Don't Know, (B) Yes, (C) No

iv. Autism could be associated with Mental Retardation?

(A) Don't Know, (B) Yes, (C) No

v. Autism could be associated with Epilepsy? (A) Don't Know, (B) Yes, (C) No

vi. Onset of Autism is usually in, (A) Neonatal age, (B) Infancy, (C) Childhood

## Appendix 2

### Scoring of Knowledge about Childhood Autism among Health Workers (KCAHW) Questionnaire

#### Domain 1

i Marked impairment in use of multiple non-verbal behaviors such as eye to eye contact, facial expression, body postures and gestures during social interaction?

(A) 0 (B) 1 (C) 0

ii Failure to develop peer relationship appropriate for developmental age?

(A) 0 (B) 1 (C) 0

iii. Lack of spontaneous will to share enjoyment, interest or activities with other people? (A) 0 (B) 1 (C) 0

iv Lack of social or emotional reciprocity? (A) 0 (B) 1 (C) 0

v Starring into open space and not focusing on any thing specific?

(A) 0 (B) 1 (C) 0

vi. The child can appear as if deaf or dumb? (A) 0 (B) 1 (C) 0

vii. Loss of interest in the environment and surroundings?

(A) 0 (B) 1 (C) 0

viii. Social smile is usually absent in a child with Autism?

(A) 0 (B) 1 (C) 0

#### Domain 2

i. Delay or total lack of development of spoken language?

(A) 0 (B) 1 (C) 0

#### Domain 3

i. Stereotyped and repetitive movement (e.g. Hand or finger flapping or twisting)?

(A) 0 (B) 1 (C) 0

ii. May be associated with abnormal eating habit?

(A) 0 (B) 1 (C) 0

iii. Persistent preoccupation with parts of objects?

(A) 0 (B) 1 (C) 0

iv. Love for regimented routine activities? (A) 0 (B) 1 (C) 0

#### Domain 4

i. Autism is Childhood Schizophrenia? (A) 0 (B) 0 (C) 1

ii. Autism is an auto-immune condition? (A) 0 (B) 0 (C) 1

iii Autism is a neuro-developmental disorder? (A) 0 (B) 1 (C) 0

iv. Autism could be associated with Mental Retardation?

(A) 0 (B) 1 (C) 0

v. Autism could be associated with Epilepsy? (A) 0 (B) 1 (C) 0

vi Onset of Autism is usually in (A) 0 (B) 0 (C) 1

A total maximum score of 19 and a minimum score of 0 are possible. The average score on the KCAHW questionnaire among a particular sample population give an index level of knowledge about childhood autism in that particular population.
